# Bis{4,4′,6,6′-tetra­chloro-2,2′-[*trans*-(*R*,*R*)-cyclo­hexane-1,2-diylbis(imino­methyl­ene)]diphenolato-κ^4^ *O*,*N*,*N*′,*O*′}zirconium(IV)

**DOI:** 10.1107/S1600536808021247

**Published:** 2008-07-16

**Authors:** Tamila Shalumova, Joseph M. Tanski

**Affiliations:** a Department of Chemistry, Vassar College, Poughkeepsie, NY 12604, USA

## Abstract

The title mononuclear complex, [Zr(C_20_H_20_Cl_4_N_2_O_2_)_2_], was obtained by allowing hexane to diffuse into a diethyl ether solution of zirconium(IV) *sec*-butoxide and the enanti­o­meri­cally pure tetra­dentate ligand *N*,*N′*-bis­(3,5-dichloro-2-hy­droxy­benz­yl)-*trans*-(*R,R*)-1,2-diamino­cyclo­hexane. The metal centre is eight-coordinate and displays a distorted dodeca­hedral coordination environment with average Zr—O and Zr—N bond lengths of 2.082 (9) and 2.441 (8) Å, respectively. In the crystal structure, complex mol­ecules are linked by inter­molecular C—H⋯Cl hydrogen-bond inter­actions into zigzag chains running parallel to the [101] direction. C—H⋯O and N—H⋯O hydrogen bonds are also present.

## Related literature

For examples of eight-coordinate zirconium complexes with related salen-type ligands (salen = *N*,*N′*-ethyl­enebis(sali­cylideneimine), see: Archer *et al.* (1979 ▶); Illingsworth *et al.* (2002 ▶); Zhu *et al.* (2005 ▶). For related literature on salan-type complexes [salan = *N,N′*-ethyl­enebis(2-hydroxy­benz­yl)], see: García-Zarracino *et al.* (2002 ▶); Yeori *et al.* (2005 ▶).
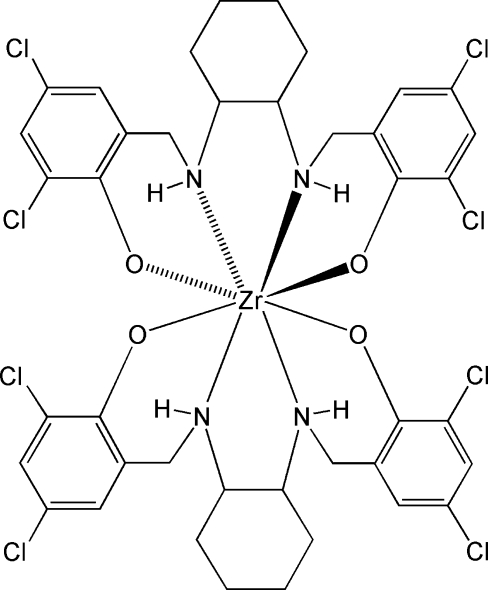


## Experimental

### 

#### Crystal data

[Zr(C_20_H_20_Cl_4_N_2_O_2_)_2_]
*M* *_r_* = 1015.58
Monoclinic, 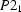

*a* = 11.2570 (6) Å
*b* = 16.4848 (8) Å
*c* = 12.7431 (6) Å
β = 114.686 (1)°
*V* = 2148.62 (18) Å^3^
*Z* = 2
Mo *K*α radiation
μ = 0.80 mm^−1^
*T* = 125 (2) K
0.15 × 0.09 × 0.02 mm

#### Data collection

Bruker APEXII CCD diffractometer
Absorption correction: multi-scan (*SADABS*; Bruker, 2007 ▶) *T*_min_ = 0.889, *T*_max_ = 0.984
29336 measured reflections
11081 independent reflections
9280 reflections with *I* > 2σ(*I*)
*R*_int_ = 0.047

#### Refinement

*R*[*F* ^2^ > 2σ(*F* ^2^)] = 0.038
*wR*(*F* ^2^) = 0.068
*S* = 1.00
11081 reflections
527 parameters
5 restraints
H atoms treated by a mixture of independent and constrained refinement
Δρ_max_ = 0.52 e Å^−3^
Δρ_min_ = −0.37 e Å^−3^
Absolute structure: Flack (1983 ▶), 5344 Friedel pairs
Flack parameter: −0.01 (3)

### 

Data collection: *APEX2* (Bruker, 2007 ▶); cell refinement: *SAINT* (Bruker, 2007 ▶); data reduction: *SAINT*; program(s) used to solve structure: *SHELXS97* (Sheldrick, 2008 ▶); program(s) used to refine structure: *SHELXL97* (Sheldrick, 2008 ▶); molecular graphics: *SHELXTL* (Sheldrick, 2008 ▶); software used to prepare material for publication: *SHELXTL*.

## Supplementary Material

Crystal structure: contains datablocks global, I. DOI: 10.1107/S1600536808021247/rz2233sup1.cif

Structure factors: contains datablocks I. DOI: 10.1107/S1600536808021247/rz2233Isup2.hkl

Additional supplementary materials:  crystallographic information; 3D view; checkCIF report

## Figures and Tables

**Table:** 

Zr—O22	2.070 (2)
Zr—O21	2.0819 (19)
Zr—O11	2.088 (2)
Zr—O12	2.089 (2)
Zr—N11	2.433 (2)
Zr—N22	2.439 (2)
Zr—N12	2.443 (2)
Zr—N21	2.451 (2)

**Table:** 

O22—Zr—O21	102.13 (8)
O22—Zr—O11	92.19 (8)
O21—Zr—O11	139.47 (8)
O22—Zr—O12	139.85 (8)
O21—Zr—O12	91.98 (8)
O11—Zr—O12	101.11 (8)
N22—Zr—N12	70.83 (8)
N11—Zr—N21	70.16 (8)

**Table 2:** Hydrogen-bond geometry (Å, °)

*D*—H⋯*A*	*D*—H	H⋯*A*	*D*⋯*A*	*D*—H⋯*A*
N11—H11⋯O22	0.89 (2)	2.28 (3)	2.662 (3)	105.7 (18)
N21—H21⋯O12	0.89 (2)	2.32 (4)	2.722 (4)	108 (2)
N21—H21⋯Cl42	0.89 (2)	2.76 (2)	3.631 (3)	166.8 (16)
N22—H22⋯O21	0.89 (2)	2.34 (3)	2.701 (4)	104.1 (18)
C72—H72*B*⋯O21	0.99	2.44	3.024 (4)	117
C132—H13*B*⋯O11	1.00	2.53	2.987 (3)	108
C141—H14*B*⋯O22	0.99	2.58	3.137 (4)	115
C51—H51*A*⋯Cl22^i^	0.95	2.83	3.654 (3)	146
C122—H12*C*⋯Cl22^ii^	0.99	2.72	3.402 (3)	126

Symmetry codes: (i) 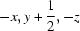; (ii) 
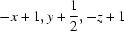
.

**Fig. 1.**
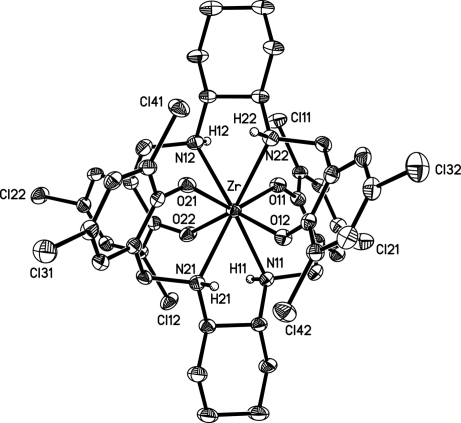
A view of the title compound, with displacement ellipsoids shown at the 50% probability level. H atoms on carbon atoms have been omitted for clarity.

## supplementary crystallographic information

### Comment

Salcean type ligands (salcean = *N,N'*-bis(2-hydroxybenzyl)-1,2-diaminocyclohexane) may be obtained by reduction of the imine C=N bond of the more ubiquitous salcen type ligands (salcen = *N,N'*-bis(salicylidene)-1,2-diaminocyclohexane), a derivative of the general class of salen ligands (salen = *N*,*N'*-ethylenebis(salicylideneimine)) containing a cyclohexyldiamine backbone (Yeori *et al.*, 2005). Whereas titanium(IV) alkoxides, Ti(OR)_4_, coordinate one salcean ligand to yield complexes of the type [salcean]Ti(OR)_2_ with the loss of two equivalents of alcohol (Yeori *et al.*, 2005), two equivalents of salcean may displace all four alkoxides from a zirconium(IV) alkoxide, resulting in a distorted dodecahedral eight-coordinate bis(salcean)zirconium(IV) complex, as reported here. Although the structure of no other bis(salcean)zirconium(IV) complex has been reported, similar eight coordinate zirconium(IV) complexes with salen type ligands exhibiting a distorted dodecahedral coordination sphere are known (Archer *et al.*, 1979; Illingsworth *et al.*, 2001; Zhu *et al., *2005).

The title compound (Fig. 1), was obtained by treating zirconium(IV) *sec*-butoxide with salcean(Cl)_4_H_4_ (salcean(Cl)_4_H_4_ = (*N,N'*-bis(3,5-dichloro-2-hydroxybenzyl)-*trans*-(*R,R*)-1,2-diamino-cyclohexane). The zirconium atom is eight-coordinate and exhibits a dodecahedral coordination geometry due to the attachment of two tetradentate salcean ligands with two O,*N*,*N'*,*O'* donor atom sets. Each salcean ligand binds the metal center in a *trans mer*-*mer* fashion (García-Zarracino *et al.*, 2002), with all four donor atoms nearly in a plane. The angle between the least-squares planes of the O,*N*,*N'*,*O'* donor set of the ligands is 87.73 (5)°. The average Zr—O bond length is 2.082 (9) Å, while the average Zr—N bond length is 2.441 (8) Å. Notable bond lengths and angles are listed in Table 1. These bond lengths and angles are similar to those reported in the literature for related bis(salen)zirconium(IV) complexes (Archer *et al.*, 1979; Zhu *et al.*, 2005). The conformation of the complex is stabilized by intramolecular N—H···O, N—H···Cl and C—H···O hydrogen bonding interactions (Table 2). In the crystal structure, complex molecules are linked into zig-zag chains running parallel to the [101] direction by intermolecular C—H···Cl hydrogen bonds (Table 2).

### Experimental

The title compound was crystallized from a 2 ml diethyl ether solution of Zr(O^S-2^Bu)_4_ (20 mg, 0.052 mmol) and *N,N'*-bis(3,5-dichloro-2-hydroxybenzyl)-*trans*-(*R,R*)-1,2-diaminocyclohexane (25 mg, 0.054 mmol), under a nitrogen atmosphere. Slow diffusion of hexanes into the solution allowed crystals to form as colourless parallelepipeds within two weeks.

### Refinement

Hydrogen atoms on carbon atoms were included in calculated positions and were refined using a riding model, with C—H = 0.95-1.00 Å and U_iso_(H) = 1.2 U_eq_(C). Hydrogen atoms on nitrogen atoms were refined semifreely with the help of a distance restraint (N—H = 0.91 Å) and with U_iso_(H) = 1.2 U_eq_(N).

### Crystal data

**Table:**

[Zr(C_20_H_20_Cl_4_N_2_O_2_)_2_]	*F*_000_ = 1032
*M**_r_* = 1015.58	*D*_x_ = 1.570 Mg m^−^^3^
Monoclinic, *P*2_1_	Mo *K*α radiation λ = 0.71073 Å
Hall symbol: P 2yb	Cell parameters from 9891 reflections
*a* = 11.2570 (6) Å	θ = 2.3–30.0º
*b* = 16.4848 (8) Å	µ = 0.80 mm^−^^1^
*c* = 12.7431 (6) Å	*T* = 125 (2) K
β = 114.6860 (10)º	Block, colourless
*V* = 2148.62 (18) Å^3^	0.15 × 0.09 × 0.02 mm
*Z* = 2	

### Data collection

**Table:**

Bruker APEXII CCD diffractometer	11081 independent reflections
Radiation source: fine-focus sealed tube	9280 reflections with *I* > 2σ(*I*)
Monochromator: graphite	*R*_int_ = 0.047
*T* = 125(2) K	θ_max_ = 28.7º
φ and ω scans	θ_min_ = 1.8º
Absorption correction: multi-scan(SADABS; Bruker, 2007)	*h* = −15→15
*T*_min_ = 0.890, *T*_max_ = 0.984	*k* = −22→22
29336 measured reflections	*l* = −17→17

### Refinement

**Table:**

Refinement on *F*^2^	Hydrogen site location: inferred from neighbouring sites
Least-squares matrix: full	H atoms treated by a mixture of independent and constrained refinement
*R*[*F*^2^ > 2σ(*F*^2^)] = 0.039	*w* = 1/[σ^2^(*F*_o_^2^) + (0.024*P*)^2^] where *P* = (*F*_o_^2^ + 2*F*_c_^2^)/3
*wR*(*F*^2^) = 0.068	(Δ/σ)_max_ < 0.001
*S* = 1.01	Δρ_max_ = 0.52 e Å^−^^3^
11081 reflections	Δρ_min_ = −0.37 e Å^−^^3^
527 parameters	Extinction correction: none
5 restraints	Absolute structure: Flack (1983), 5344 Friedel pairs
Primary atom site location: structure-invariant direct methods	Flack parameter: −0.01 (3)
Secondary atom site location: difference Fourier map	

### Special details

**Table:**

Geometry. All e.s.d.'s (except the e.s.d. in the dihedral angle between two l.s. planes) are estimated using the full covariance matrix. The cell e.s.d.'s are taken into account individually in the estimation of e.s.d.'s in distances, angles and torsion angles; correlations between e.s.d.'s in cell parameters are only used when they are defined by crystal symmetry. An approximate (isotropic) treatment of cell e.s.d.'s is used for estimating e.s.d.'s involving l.s. planes.
Refinement. Refinement of *F*^2^ against ALL reflections. The weighted *R*-factor *wR* and goodness of fit *S* are based on *F*^2^, conventional *R*-factors *R* are based on *F*, with *F* set to zero for negative *F*^2^. The threshold expression of *F*^2^ > σ(*F*^2^) is used only for calculating *R*-factors(gt) *etc*. and is not relevant to the choice of reflections for refinement. *R*-factors based on *F*^2^ are statistically about twice as large as those based on *F*, and *R*-factors based on ALL data will be even larger. EXTI refined to zero and was removed from the refinement.

### Fractional atomic coordinates and isotropic or equivalent isotropic displacement parameters (Å^2^)

**Table:**

	*x*	*y*	*z*	*U*_iso_*/*U*_eq_	
Zr	0.21852 (2)	0.025696 (16)	0.24648 (2)	0.01679 (6)	
O11	0.06724 (19)	−0.02645 (12)	0.27759 (17)	0.0217 (5)	
O12	0.16648 (19)	0.14816 (12)	0.23235 (16)	0.0209 (4)	
O21	0.41628 (18)	0.05315 (12)	0.33466 (16)	0.0220 (5)	
O22	0.2219 (2)	−0.07046 (12)	0.14332 (17)	0.0239 (5)	
N11	0.0281 (2)	0.03706 (14)	0.06275 (19)	0.0177 (5)	
H11	0.023 (3)	−0.0137 (12)	0.037 (2)	0.021*	
N12	0.3180 (2)	−0.08819 (15)	0.3763 (2)	0.0204 (5)	
H12	0.252 (2)	−0.1231 (16)	0.362 (3)	0.025*	
N21	0.2823 (2)	0.08477 (16)	0.1011 (2)	0.0192 (5)	
H21	0.283 (3)	0.1366 (11)	0.120 (3)	0.023*	
N22	0.2551 (2)	0.06397 (15)	0.4425 (2)	0.0197 (5)	
H22	0.334 (2)	0.0871 (17)	0.470 (2)	0.024*	
Cl11	0.01552 (7)	−0.17386 (5)	0.38480 (6)	0.02992 (19)	
Cl12	0.09931 (8)	−0.14904 (5)	−0.08304 (6)	0.03103 (19)	
Cl21	−0.45928 (8)	−0.16006 (6)	0.02610 (7)	0.0382 (2)	
Cl22	0.44983 (8)	−0.38479 (5)	0.12259 (7)	0.03275 (19)	
Cl31	0.85232 (8)	0.22030 (5)	0.29128 (7)	0.0344 (2)	
Cl32	0.23253 (9)	0.43591 (5)	0.52203 (8)	0.0388 (2)	
Cl41	0.60590 (7)	0.12385 (5)	0.55203 (6)	0.02962 (18)	
Cl42	0.22922 (8)	0.29830 (5)	0.13700 (7)	0.03235 (19)	
C11	−0.0509 (3)	−0.05738 (18)	0.2207 (2)	0.0195 (6)	
C12	0.2697 (3)	−0.14329 (18)	0.1422 (2)	0.0183 (6)	
C21	−0.0926 (3)	−0.12690 (18)	0.2586 (2)	0.0211 (7)	
C22	0.2245 (3)	−0.18855 (18)	0.0405 (2)	0.0210 (6)	
C31	−0.2177 (3)	−0.15817 (19)	0.2006 (2)	0.0246 (7)	
H31A	−0.2445	−0.2046	0.2293	0.029*	
C32	0.2770 (3)	−0.26272 (18)	0.0334 (2)	0.0208 (6)	
H32A	0.2453	−0.2922	−0.0371	0.025*	
C41	−0.3018 (3)	−0.12093 (19)	0.1011 (3)	0.0244 (7)	
C42	0.3770 (3)	−0.29308 (18)	0.1317 (3)	0.0222 (7)	
C51	−0.2648 (3)	−0.05332 (19)	0.0580 (3)	0.0236 (7)	
H51A	−0.3239	−0.0292	−0.0121	0.028*	
C52	0.4222 (3)	−0.25144 (18)	0.2346 (3)	0.0214 (7)	
H52A	0.4900	−0.2739	0.3015	0.026*	
C61	−0.1398 (3)	−0.02071 (17)	0.1184 (3)	0.0194 (6)	
C62	0.3691 (3)	−0.17704 (18)	0.2409 (2)	0.0198 (6)	
C71	−0.0945 (3)	0.05371 (17)	0.0772 (3)	0.0210 (7)	
H71A	−0.1637	0.0716	0.0025	0.025*	
H71B	−0.0788	0.0981	0.1338	0.025*	
C72	0.4216 (3)	−0.12984 (19)	0.3524 (3)	0.0251 (7)	
H72A	0.4700	−0.1673	0.4169	0.030*	
H72B	0.4843	−0.0887	0.3495	0.030*	
C81	0.0454 (3)	0.09205 (19)	−0.0218 (2)	0.0214 (7)	
H81A	0.0416	0.1494	0.0024	0.026*	
C82	0.3656 (3)	−0.06773 (18)	0.5012 (2)	0.0209 (6)	
H82A	0.4527	−0.0408	0.5260	0.025*	
C91	−0.0596 (3)	0.0805 (2)	−0.1450 (2)	0.0300 (8)	
H91A	−0.0592	0.0233	−0.1686	0.036*	
H91B	−0.1466	0.0921	−0.1466	0.036*	
C92	0.3835 (3)	−0.1424 (2)	0.5775 (2)	0.0296 (7)	
H92A	0.4472	−0.1796	0.5678	0.036*	
H92B	0.2990	−0.1714	0.5523	0.036*	
C101	−0.0371 (3)	0.1360 (2)	−0.2309 (3)	0.0399 (10)	
H10A	−0.1039	0.1250	−0.3099	0.048*	
H10B	−0.0459	0.1933	−0.2121	0.048*	
C102	0.4318 (4)	−0.1200 (2)	0.7053 (3)	0.0346 (8)	
H10C	0.4367	−0.1695	0.7510	0.041*	
H10D	0.5207	−0.0965	0.7333	0.041*	
C111	0.0985 (3)	0.1222 (2)	−0.2266 (3)	0.0330 (8)	
H11A	0.1136	0.1607	−0.2794	0.040*	
H11B	0.1042	0.0665	−0.2530	0.040*	
C112	0.3401 (3)	−0.0589 (2)	0.7229 (3)	0.0343 (8)	
H11C	0.3760	−0.0421	0.8049	0.041*	
H11D	0.2537	−0.0842	0.7030	0.041*	
C121	0.2032 (3)	0.1340 (2)	−0.1045 (3)	0.0277 (7)	
H12A	0.2015	0.1909	−0.0803	0.033*	
H12B	0.2903	0.1235	−0.1034	0.033*	
C122	0.3248 (3)	0.0153 (2)	0.6464 (2)	0.0268 (7)	
H12C	0.4107	0.0422	0.6702	0.032*	
H12D	0.2643	0.0543	0.6577	0.032*	
C131	0.1815 (3)	0.07684 (19)	−0.0196 (2)	0.0210 (6)	
H13A	0.1839	0.0199	−0.0455	0.025*	
C132	0.2722 (3)	−0.00701 (17)	0.5183 (2)	0.0203 (6)	
H13B	0.1853	−0.0339	0.4953	0.024*	
C141	0.4133 (3)	0.05631 (19)	0.1134 (2)	0.0212 (6)	
H14A	0.4247	0.0699	0.0425	0.025*	
H14B	0.4186	−0.0034	0.1227	0.025*	
C142	0.1574 (3)	0.12355 (18)	0.4452 (2)	0.0219 (6)	
H14C	0.1619	0.1267	0.5243	0.026*	
H14D	0.0684	0.1054	0.3927	0.026*	
C151	0.5211 (3)	0.09539 (18)	0.2165 (2)	0.0190 (6)	
C152	0.1832 (3)	0.20643 (18)	0.4083 (3)	0.0207 (6)	
C161	0.6252 (3)	0.13470 (18)	0.2066 (3)	0.0231 (7)	
H16A	0.6290	0.1382	0.1336	0.028*	
C162	0.1994 (3)	0.27479 (19)	0.4771 (3)	0.0243 (7)	
H16B	0.1965	0.2701	0.5503	0.029*	
C171	0.7234 (3)	0.16867 (18)	0.3034 (3)	0.0227 (7)	
C172	0.2197 (3)	0.34961 (19)	0.4383 (3)	0.0264 (7)	
C181	0.7206 (3)	0.16425 (18)	0.4112 (3)	0.0214 (6)	
H18A	0.7894	0.1864	0.4777	0.026*	
C182	0.2297 (3)	0.35755 (19)	0.3345 (3)	0.0268 (7)	
H18B	0.2460	0.4090	0.3095	0.032*	
C192	0.2155 (3)	0.28884 (19)	0.2676 (2)	0.0226 (7)	
C191	0.6144 (3)	0.12660 (18)	0.4186 (2)	0.0212 (6)	
C201	0.5142 (3)	0.09082 (17)	0.3237 (2)	0.0185 (6)	
C202	0.1886 (3)	0.21253 (18)	0.3006 (2)	0.0185 (6)	

### Atomic displacement parameters (Å^2^)

**Table:**

	*U*^11^	*U*^22^	*U*^33^	*U*^12^	*U*^13^	*U*^23^
Zr	0.01607 (13)	0.01881 (13)	0.01375 (12)	−0.00159 (13)	0.00449 (10)	−0.00096 (13)
O11	0.0170 (11)	0.0270 (12)	0.0191 (11)	−0.0034 (9)	0.0057 (9)	0.0011 (9)
O12	0.0244 (11)	0.0181 (11)	0.0202 (11)	−0.0019 (9)	0.0092 (9)	−0.0017 (9)
O21	0.0191 (11)	0.0292 (12)	0.0160 (10)	−0.0038 (9)	0.0056 (9)	−0.0015 (8)
O22	0.0262 (11)	0.0224 (11)	0.0169 (10)	0.0033 (9)	0.0030 (9)	−0.0027 (9)
N11	0.0180 (11)	0.0157 (14)	0.0185 (12)	−0.0003 (10)	0.0067 (9)	−0.0002 (10)
N12	0.0198 (13)	0.0215 (13)	0.0160 (13)	−0.0021 (10)	0.0035 (11)	−0.0017 (10)
N21	0.0180 (13)	0.0216 (13)	0.0164 (12)	−0.0008 (11)	0.0057 (10)	−0.0019 (11)
N22	0.0189 (13)	0.0218 (14)	0.0188 (13)	−0.0046 (10)	0.0082 (11)	−0.0023 (11)
Cl11	0.0262 (4)	0.0322 (4)	0.0247 (4)	−0.0048 (3)	0.0040 (3)	0.0092 (3)
Cl12	0.0278 (4)	0.0327 (4)	0.0206 (4)	0.0081 (3)	−0.0017 (3)	−0.0056 (3)
Cl21	0.0245 (4)	0.0513 (6)	0.0302 (5)	−0.0175 (4)	0.0028 (3)	0.0061 (4)
Cl22	0.0401 (5)	0.0262 (4)	0.0280 (4)	0.0121 (4)	0.0103 (4)	0.0001 (3)
Cl31	0.0229 (4)	0.0460 (5)	0.0328 (5)	−0.0120 (4)	0.0101 (4)	0.0010 (4)
Cl32	0.0415 (5)	0.0266 (4)	0.0482 (6)	−0.0051 (4)	0.0188 (4)	−0.0158 (4)
Cl41	0.0254 (4)	0.0451 (5)	0.0175 (4)	−0.0067 (4)	0.0080 (3)	−0.0077 (3)
Cl42	0.0419 (5)	0.0283 (4)	0.0325 (5)	−0.0046 (4)	0.0211 (4)	0.0028 (4)
C11	0.0208 (15)	0.0200 (15)	0.0180 (15)	−0.0033 (12)	0.0084 (13)	−0.0026 (12)
C12	0.0173 (15)	0.0204 (15)	0.0171 (14)	0.0018 (12)	0.0071 (12)	−0.0005 (12)
C21	0.0202 (16)	0.0260 (17)	0.0158 (15)	0.0001 (13)	0.0061 (13)	0.0016 (12)
C22	0.0155 (14)	0.0275 (17)	0.0167 (15)	0.0010 (12)	0.0035 (12)	0.0018 (13)
C31	0.0308 (17)	0.0248 (17)	0.0210 (16)	−0.0056 (14)	0.0136 (14)	−0.0018 (13)
C32	0.0219 (16)	0.0215 (16)	0.0193 (15)	−0.0026 (13)	0.0088 (13)	−0.0046 (13)
C41	0.0187 (16)	0.0303 (18)	0.0216 (16)	−0.0068 (13)	0.0058 (13)	−0.0044 (14)
C42	0.0241 (16)	0.0194 (16)	0.0245 (16)	0.0040 (13)	0.0116 (14)	0.0026 (13)
C51	0.0192 (15)	0.0298 (18)	0.0200 (15)	−0.0005 (13)	0.0065 (13)	0.0001 (13)
C52	0.0204 (16)	0.0249 (16)	0.0168 (15)	−0.0010 (13)	0.0056 (13)	0.0034 (13)
C61	0.0194 (15)	0.0190 (16)	0.0209 (16)	−0.0006 (12)	0.0094 (13)	−0.0004 (12)
C62	0.0193 (15)	0.0219 (16)	0.0168 (14)	0.0003 (12)	0.0062 (12)	−0.0001 (12)
C71	0.0157 (15)	0.0219 (16)	0.0217 (15)	0.0018 (12)	0.0040 (12)	0.0011 (12)
C72	0.0230 (17)	0.0247 (17)	0.0228 (16)	0.0042 (13)	0.0048 (13)	−0.0010 (13)
C81	0.0172 (15)	0.0286 (17)	0.0170 (15)	−0.0004 (12)	0.0059 (12)	0.0037 (13)
C82	0.0225 (16)	0.0240 (16)	0.0130 (14)	−0.0012 (13)	0.0043 (12)	−0.0006 (13)
C91	0.0165 (16)	0.050 (2)	0.0180 (16)	0.0031 (15)	0.0020 (13)	0.0077 (15)
C92	0.039 (2)	0.0280 (18)	0.0163 (16)	0.0026 (15)	0.0064 (14)	0.0046 (14)
C101	0.0252 (18)	0.070 (3)	0.0229 (18)	0.0049 (18)	0.0084 (15)	0.0179 (18)
C102	0.041 (2)	0.038 (2)	0.0208 (17)	0.0012 (16)	0.0091 (16)	0.0085 (15)
C111	0.0260 (18)	0.053 (2)	0.0192 (17)	−0.0008 (17)	0.0081 (14)	0.0090 (16)
C112	0.041 (2)	0.043 (2)	0.0170 (16)	0.0005 (17)	0.0095 (15)	0.0035 (15)
C121	0.0235 (17)	0.038 (2)	0.0206 (16)	−0.0022 (14)	0.0083 (13)	0.0055 (14)
C122	0.0312 (16)	0.0317 (19)	0.0149 (14)	−0.0029 (15)	0.0071 (12)	−0.0014 (14)
C131	0.0178 (15)	0.0281 (17)	0.0148 (14)	−0.0023 (13)	0.0045 (12)	−0.0008 (13)
C132	0.0234 (16)	0.0210 (15)	0.0159 (15)	−0.0027 (12)	0.0077 (13)	0.0019 (12)
C141	0.0159 (14)	0.0341 (17)	0.0132 (14)	−0.0016 (12)	0.0056 (12)	−0.0017 (12)
C142	0.0239 (16)	0.0243 (16)	0.0184 (15)	−0.0001 (13)	0.0098 (13)	−0.0018 (13)
C151	0.0134 (14)	0.0244 (16)	0.0174 (15)	−0.0006 (12)	0.0045 (12)	−0.0008 (12)
C152	0.0161 (15)	0.0218 (16)	0.0239 (16)	−0.0006 (12)	0.0080 (13)	−0.0001 (13)
C161	0.0215 (16)	0.0302 (18)	0.0183 (15)	0.0023 (13)	0.0089 (13)	0.0006 (13)
C162	0.0191 (16)	0.0272 (18)	0.0264 (17)	0.0005 (13)	0.0094 (14)	−0.0031 (14)
C171	0.0162 (15)	0.0236 (17)	0.0272 (17)	−0.0042 (13)	0.0081 (13)	0.0016 (13)
C172	0.0202 (16)	0.0270 (17)	0.0290 (17)	−0.0016 (14)	0.0072 (13)	−0.0102 (14)
C181	0.0178 (15)	0.0208 (15)	0.0203 (15)	−0.0005 (12)	0.0026 (12)	−0.0021 (13)
C182	0.0228 (16)	0.0199 (16)	0.0354 (19)	−0.0052 (13)	0.0099 (14)	−0.0017 (14)
C192	0.0194 (15)	0.0270 (17)	0.0219 (16)	−0.0008 (13)	0.0089 (13)	0.0014 (13)
C191	0.0232 (16)	0.0226 (16)	0.0169 (14)	0.0013 (13)	0.0075 (12)	0.0015 (13)
C201	0.0161 (15)	0.0189 (15)	0.0195 (15)	0.0022 (12)	0.0064 (12)	0.0012 (12)
C202	0.0124 (14)	0.0207 (16)	0.0196 (15)	−0.0014 (12)	0.0039 (12)	−0.0015 (12)

### Geometric parameters (Å, °)

**Table:**

Zr—O22	2.070 (2)	C81—C91	1.533 (4)
Zr—O21	2.0819 (19)	C81—C131	1.541 (4)
Zr—O11	2.088 (2)	C81—H81A	1.0000
Zr—O12	2.089 (2)	C82—C92	1.529 (4)
Zr—N11	2.433 (2)	C82—C132	1.532 (4)
Zr—N22	2.439 (2)	C82—H82A	1.0000
Zr—N12	2.443 (2)	C91—C101	1.527 (4)
Zr—N21	2.451 (2)	C91—H91A	0.9900
O11—C11	1.322 (3)	C91—H91B	0.9900
O12—C202	1.328 (3)	C92—C102	1.531 (4)
O21—C201	1.323 (3)	C92—H92A	0.9900
O22—C12	1.318 (3)	C92—H92B	0.9900
N11—C81	1.482 (3)	C101—C111	1.521 (4)
N11—C71	1.492 (4)	C101—H10A	0.9900
N11—H11	0.892 (17)	C101—H10B	0.9900
N12—C82	1.491 (4)	C102—C112	1.524 (5)
N12—C72	1.491 (4)	C102—H10C	0.9900
N12—H12	0.900 (17)	C102—H10D	0.9900
N21—C131	1.487 (3)	C111—C121	1.521 (4)
N21—C141	1.492 (4)	C111—H11A	0.9900
N21—H21	0.885 (17)	C111—H11B	0.9900
N22—C132	1.478 (4)	C112—C122	1.528 (4)
N22—C142	1.486 (4)	C112—H11C	0.9900
N22—H22	0.896 (17)	C112—H11D	0.9900
Cl11—C21	1.741 (3)	C121—C131	1.529 (4)
Cl12—C22	1.742 (3)	C121—H12A	0.9900
Cl21—C41	1.749 (3)	C121—H12B	0.9900
Cl22—C42	1.746 (3)	C122—C132	1.530 (4)
Cl31—C171	1.743 (3)	C122—H12C	0.9900
Cl32—C172	1.748 (3)	C122—H12D	0.9900
Cl41—C191	1.744 (3)	C131—H13A	1.0000
Cl42—C192	1.742 (3)	C132—H13B	1.0000
C11—C21	1.399 (4)	C141—C151	1.511 (4)
C11—C61	1.406 (4)	C141—H14A	0.9900
C12—C22	1.395 (4)	C141—H14B	0.9900
C12—C62	1.403 (4)	C142—C152	1.512 (4)
C21—C31	1.388 (4)	C142—H14C	0.9900
C22—C32	1.377 (4)	C142—H14D	0.9900
C31—C41	1.371 (4)	C151—C161	1.391 (4)
C31—H31A	0.9500	C151—C201	1.403 (4)
C32—C42	1.382 (4)	C152—C162	1.392 (4)
C32—H32A	0.9500	C152—C202	1.403 (4)
C41—C51	1.381 (4)	C161—C171	1.385 (4)
C42—C52	1.376 (4)	C161—H16A	0.9500
C51—C61	1.399 (4)	C162—C172	1.383 (4)
C51—H51A	0.9500	C162—H16B	0.9500
C52—C62	1.381 (4)	C171—C181	1.389 (4)
C52—H52A	0.9500	C172—C182	1.379 (4)
C61—C71	1.505 (4)	C181—C191	1.385 (4)
C62—C72	1.507 (4)	C181—H18A	0.9500
C71—H71A	0.9900	C182—C192	1.386 (4)
C71—H71B	0.9900	C182—H18B	0.9500
C72—H72A	0.9900	C192—C202	1.399 (4)
C72—H72B	0.9900	C191—C201	1.393 (4)
			
O22—Zr—O21	102.13 (8)	C92—C82—C132	110.5 (2)
O22—Zr—O11	92.19 (8)	N12—C82—H82A	107.8
O21—Zr—O11	139.47 (8)	C92—C82—H82A	107.8
O22—Zr—O12	139.85 (8)	C132—C82—H82A	107.8
O21—Zr—O12	91.98 (8)	C101—C91—C81	111.9 (3)
O11—Zr—O12	101.11 (8)	C101—C91—H91A	109.2
O22—Zr—N11	71.96 (8)	C81—C91—H91A	109.2
O21—Zr—N11	144.73 (8)	C101—C91—H91B	109.2
O11—Zr—N11	75.75 (7)	C81—C91—H91B	109.2
O12—Zr—N11	74.93 (8)	H91A—C91—H91B	107.9
O22—Zr—N22	143.96 (8)	C82—C92—C102	112.1 (3)
O21—Zr—N22	72.90 (8)	C82—C92—H92A	109.2
O11—Zr—N22	73.39 (8)	C102—C92—H92A	109.2
O12—Zr—N22	76.09 (8)	C82—C92—H92B	109.2
N11—Zr—N22	132.04 (8)	C102—C92—H92B	109.2
O22—Zr—N12	73.55 (8)	H92A—C92—H92B	107.9
O21—Zr—N12	73.57 (8)	C111—C101—C91	110.4 (3)
O11—Zr—N12	74.61 (8)	C111—C101—H10A	109.6
O12—Zr—N12	146.50 (8)	C91—C101—H10A	109.6
N11—Zr—N12	132.94 (8)	C111—C101—H10B	109.6
N22—Zr—N12	70.83 (8)	C91—C101—H10B	109.6
O22—Zr—N21	74.65 (8)	H10A—C101—H10B	108.1
O21—Zr—N21	74.74 (8)	C112—C102—C92	110.7 (3)
O11—Zr—N21	145.76 (8)	C112—C102—H10C	109.5
O12—Zr—N21	73.20 (8)	C92—C102—H10C	109.5
N11—Zr—N21	70.16 (8)	C112—C102—H10D	109.5
N22—Zr—N21	133.95 (8)	C92—C102—H10D	109.5
N12—Zr—N21	128.39 (8)	H10C—C102—H10D	108.1
C11—O11—Zr	140.16 (18)	C101—C111—C121	110.8 (3)
C202—O12—Zr	138.32 (18)	C101—C111—H11A	109.5
C201—O21—Zr	142.74 (18)	C121—C111—H11A	109.5
C12—O22—Zr	145.11 (18)	C101—C111—H11B	109.5
C81—N11—C71	112.7 (2)	C121—C111—H11B	109.5
C81—N11—Zr	114.59 (16)	H11A—C111—H11B	108.1
C71—N11—Zr	112.57 (16)	C102—C112—C122	109.8 (3)
C81—N11—H11	109 (2)	C102—C112—H11C	109.7
C71—N11—H11	107 (2)	C122—C112—H11C	109.7
Zr—N11—H11	100.3 (19)	C102—C112—H11D	109.7
C82—N12—C72	111.1 (2)	C122—C112—H11D	109.7
C82—N12—Zr	114.07 (18)	H11C—C112—H11D	108.2
C72—N12—Zr	113.07 (18)	C111—C121—C131	111.1 (3)
C82—N12—H12	105 (2)	C111—C121—H12A	109.4
C72—N12—H12	109 (2)	C131—C121—H12A	109.4
Zr—N12—H12	105 (2)	C111—C121—H12B	109.4
C131—N21—C141	112.0 (2)	C131—C121—H12B	109.4
C131—N21—Zr	114.14 (17)	H12A—C121—H12B	108.0
C141—N21—Zr	112.61 (17)	C112—C122—C132	112.3 (3)
C131—N21—H21	106 (2)	C112—C122—H12C	109.2
C141—N21—H21	112 (2)	C132—C122—H12C	109.2
Zr—N21—H21	99 (2)	C112—C122—H12D	109.2
C132—N22—C142	113.8 (2)	C132—C122—H12D	109.2
C132—N22—Zr	112.64 (17)	H12C—C122—H12D	107.9
C142—N22—Zr	112.62 (17)	N21—C131—C121	113.5 (2)
C132—N22—H22	104 (2)	N21—C131—C81	109.1 (2)
C142—N22—H22	110 (2)	C121—C131—C81	109.8 (2)
Zr—N22—H22	103.1 (19)	N21—C131—H13A	108.1
O11—C11—C21	122.1 (3)	C121—C131—H13A	108.1
O11—C11—C61	120.4 (3)	C81—C131—H13A	108.1
C21—C11—C61	117.5 (3)	N22—C132—C122	113.2 (2)
O22—C12—C22	120.4 (2)	N22—C132—C82	109.5 (2)
O22—C12—C62	121.9 (3)	C122—C132—C82	109.0 (2)
C22—C12—C62	117.8 (3)	N22—C132—H13B	108.3
C31—C21—C11	122.2 (3)	C122—C132—H13B	108.3
C31—C21—Cl11	119.3 (2)	C82—C132—H13B	108.3
C11—C21—Cl11	118.5 (2)	N21—C141—C151	110.9 (2)
C32—C22—C12	122.4 (3)	N21—C141—H14A	109.5
C32—C22—Cl12	118.7 (2)	C151—C141—H14A	109.5
C12—C22—Cl12	118.9 (2)	N21—C141—H14B	109.5
C41—C31—C21	118.8 (3)	C151—C141—H14B	109.5
C41—C31—H31A	120.6	H14A—C141—H14B	108.0
C21—C31—H31A	120.6	N22—C142—C152	110.5 (2)
C22—C32—C42	118.1 (3)	N22—C142—H14C	109.5
C22—C32—H32A	120.9	C152—C142—H14C	109.5
C42—C32—H32A	120.9	N22—C142—H14D	109.5
C31—C41—C51	121.5 (3)	C152—C142—H14D	109.5
C31—C41—Cl21	119.4 (2)	H14C—C142—H14D	108.1
C51—C41—Cl21	119.0 (2)	C161—C151—C201	120.3 (3)
C52—C42—C32	121.3 (3)	C161—C151—C141	121.4 (3)
C52—C42—Cl22	119.9 (2)	C201—C151—C141	118.3 (3)
C32—C42—Cl22	118.7 (2)	C162—C152—C202	120.7 (3)
C41—C51—C61	119.5 (3)	C162—C152—C142	121.8 (3)
C41—C51—H51A	120.3	C202—C152—C142	117.5 (3)
C61—C51—H51A	120.3	C171—C161—C151	119.8 (3)
C42—C52—C62	120.1 (3)	C171—C161—H16A	120.1
C42—C52—H52A	119.9	C151—C161—H16A	120.1
C62—C52—H52A	119.9	C172—C162—C152	119.6 (3)
C51—C61—C11	120.5 (3)	C172—C162—H16B	120.2
C51—C61—C71	122.1 (3)	C152—C162—H16B	120.2
C11—C61—C71	117.4 (3)	C161—C171—C181	121.3 (3)
C52—C62—C12	120.2 (3)	C161—C171—Cl31	120.2 (2)
C52—C62—C72	120.3 (3)	C181—C171—Cl31	118.5 (2)
C12—C62—C72	119.5 (3)	C182—C172—C162	121.3 (3)
N11—C71—C61	111.0 (2)	C182—C172—Cl32	119.3 (3)
N11—C71—H71A	109.4	C162—C172—Cl32	119.4 (2)
C61—C71—H71A	109.4	C191—C181—C171	117.9 (3)
N11—C71—H71B	109.4	C191—C181—H18A	121.1
C61—C71—H71B	109.4	C171—C181—H18A	121.1
H71A—C71—H71B	108.0	C172—C182—C192	118.5 (3)
N12—C72—C62	113.5 (2)	C172—C182—H18B	120.7
N12—C72—H72A	108.9	C192—C182—H18B	120.7
C62—C72—H72A	108.9	C182—C192—C202	122.3 (3)
N12—C72—H72B	108.9	C182—C192—Cl42	118.8 (2)
C62—C72—H72B	108.9	C202—C192—Cl42	118.9 (2)
H72A—C72—H72B	107.7	C181—C191—C201	122.7 (3)
N11—C81—C91	112.9 (2)	C181—C191—Cl41	118.6 (2)
N11—C81—C131	108.6 (2)	C201—C191—Cl41	118.7 (2)
C91—C81—C131	109.5 (2)	O21—C201—C191	121.1 (3)
N11—C81—H81A	108.6	O21—C201—C151	121.0 (3)
C91—C81—H81A	108.6	C191—C201—C151	117.9 (3)
C131—C81—H81A	108.6	O12—C202—C192	121.8 (3)
N12—C82—C92	113.0 (2)	O12—C202—C152	120.7 (3)
N12—C82—C132	109.7 (2)	C192—C202—C152	117.5 (3)
			
O22—Zr—O11—C11	−52.3 (3)	Cl21—C41—C51—C61	178.9 (2)
O21—Zr—O11—C11	−163.9 (3)	C32—C42—C52—C62	−1.1 (5)
O12—Zr—O11—C11	89.6 (3)	Cl22—C42—C52—C62	176.9 (2)
N11—Zr—O11—C11	18.5 (3)	C41—C51—C61—C11	1.6 (4)
N22—Zr—O11—C11	161.3 (3)	C41—C51—C61—C71	−178.9 (3)
N12—Zr—O11—C11	−124.6 (3)	O11—C11—C61—C51	180.0 (3)
N21—Zr—O11—C11	13.2 (4)	C21—C11—C61—C51	0.0 (4)
O22—Zr—O12—C202	−162.5 (2)	O11—C11—C61—C71	0.4 (4)
O21—Zr—O12—C202	−50.9 (3)	C21—C11—C61—C71	−179.6 (3)
O11—Zr—O12—C202	90.5 (3)	C42—C52—C62—C12	−0.5 (4)
N11—Zr—O12—C202	162.3 (3)	C42—C52—C62—C72	−177.7 (3)
N22—Zr—O12—C202	20.9 (3)	O22—C12—C62—C52	−176.0 (3)
N12—Zr—O12—C202	11.6 (3)	C22—C12—C62—C52	2.1 (4)
N21—Zr—O12—C202	−124.3 (3)	O22—C12—C62—C72	1.2 (4)
O22—Zr—O21—C201	79.1 (3)	C22—C12—C62—C72	179.4 (3)
O11—Zr—O21—C201	−172.8 (3)	C81—N11—C71—C61	155.0 (2)
O12—Zr—O21—C201	−63.0 (3)	Zr—N11—C71—C61	−73.5 (2)
N11—Zr—O21—C201	3.3 (4)	C51—C61—C71—N11	−123.8 (3)
N22—Zr—O21—C201	−137.8 (3)	C11—C61—C71—N11	55.7 (3)
N12—Zr—O21—C201	147.7 (3)	C82—N12—C72—C62	160.1 (2)
N21—Zr—O21—C201	8.9 (3)	Zr—N12—C72—C62	−70.2 (3)
O21—Zr—O22—C12	55.4 (3)	C52—C62—C72—N12	−140.9 (3)
O11—Zr—O22—C12	−86.4 (3)	C12—C62—C72—N12	41.9 (4)
O12—Zr—O22—C12	163.4 (3)	C71—N11—C81—C91	−65.0 (3)
N11—Zr—O22—C12	−160.7 (3)	Zr—N11—C81—C91	164.5 (2)
N22—Zr—O22—C12	−22.1 (4)	C71—N11—C81—C131	173.3 (2)
N12—Zr—O22—C12	−13.2 (3)	Zr—N11—C81—C131	42.8 (3)
N21—Zr—O22—C12	125.7 (3)	C72—N12—C82—C92	−70.5 (3)
O22—Zr—N11—C81	−96.17 (19)	Zr—N12—C82—C92	160.3 (2)
O21—Zr—N11—C81	−10.6 (3)	C72—N12—C82—C132	165.7 (2)
O11—Zr—N11—C81	166.7 (2)	Zr—N12—C82—C132	36.5 (3)
O12—Zr—N11—C81	60.78 (18)	N11—C81—C91—C101	−178.2 (3)
N22—Zr—N11—C81	115.47 (19)	C131—C81—C91—C101	−57.1 (4)
N12—Zr—N11—C81	−140.93 (18)	N12—C82—C92—C102	−179.7 (3)
N21—Zr—N11—C81	−16.45 (18)	C132—C82—C92—C102	−56.3 (4)
O22—Zr—N11—C71	133.25 (19)	C81—C91—C101—C111	56.4 (4)
O21—Zr—N11—C71	−141.21 (17)	C82—C92—C102—C112	55.6 (4)
O11—Zr—N11—C71	36.12 (17)	C91—C101—C111—C121	−55.9 (4)
O12—Zr—N11—C71	−69.80 (18)	C92—C102—C112—C122	−55.3 (4)
N22—Zr—N11—C71	−15.1 (2)	C101—C111—C121—C131	57.8 (4)
N12—Zr—N11—C71	88.5 (2)	C102—C112—C122—C132	58.2 (4)
N21—Zr—N11—C71	−147.0 (2)	C141—N21—C131—C121	−67.6 (3)
O22—Zr—N12—C82	175.8 (2)	Zr—N21—C131—C121	163.0 (2)
O21—Zr—N12—C82	67.38 (19)	C141—N21—C131—C81	169.6 (2)
O11—Zr—N12—C82	−87.2 (2)	Zr—N21—C131—C81	40.2 (3)
O12—Zr—N12—C82	−0.3 (3)	C111—C121—C131—N21	179.1 (3)
N11—Zr—N12—C82	−139.96 (18)	C111—C121—C131—C81	−58.4 (3)
N22—Zr—N12—C82	−9.79 (18)	N11—C81—C131—N21	−53.9 (3)
N21—Zr—N12—C82	121.66 (19)	C91—C81—C131—N21	−177.7 (2)
O22—Zr—N12—C72	47.57 (18)	N11—C81—C131—C121	−179.0 (2)
O21—Zr—N12—C72	−60.81 (18)	C91—C81—C131—C121	57.3 (3)
O11—Zr—N12—C72	144.6 (2)	C142—N22—C132—C122	−63.0 (3)
O12—Zr—N12—C72	−128.47 (19)	Zr—N22—C132—C122	167.24 (19)
N11—Zr—N12—C72	91.8 (2)	C142—N22—C132—C82	175.2 (2)
N22—Zr—N12—C72	−138.0 (2)	Zr—N22—C132—C82	45.4 (3)
N21—Zr—N12—C72	−6.5 (2)	C112—C122—C132—N22	179.3 (3)
O22—Zr—N21—C131	62.43 (19)	C112—C122—C132—C82	−58.5 (3)
O21—Zr—N21—C131	169.9 (2)	N12—C82—C132—N22	−53.9 (3)
O11—Zr—N21—C131	−8.1 (3)	C92—C82—C132—N22	−179.2 (2)
O12—Zr—N21—C131	−93.19 (19)	N12—C82—C132—C122	−178.3 (2)
N11—Zr—N21—C131	−13.55 (18)	C92—C82—C132—C122	56.5 (3)
N22—Zr—N21—C131	−143.41 (18)	C131—N21—C141—C151	156.1 (2)
N12—Zr—N21—C131	116.11 (19)	Zr—N21—C141—C151	−73.7 (3)
O22—Zr—N21—C141	−66.70 (18)	C132—N22—C142—C152	156.8 (2)
O21—Zr—N21—C141	40.80 (18)	Zr—N22—C142—C152	−73.4 (2)
O11—Zr—N21—C141	−137.25 (18)	N21—C141—C151—C161	−128.1 (3)
O12—Zr—N21—C141	137.68 (19)	N21—C141—C151—C201	52.3 (4)
N11—Zr—N21—C141	−142.7 (2)	N22—C142—C152—C162	−125.4 (3)
N22—Zr—N21—C141	87.5 (2)	N22—C142—C152—C202	55.4 (3)
N12—Zr—N21—C141	−13.0 (2)	C201—C151—C161—C171	0.7 (4)
O22—Zr—N22—C132	−10.3 (3)	C141—C151—C161—C171	−178.9 (3)
O21—Zr—N22—C132	−97.45 (19)	C202—C152—C162—C172	0.7 (4)
O11—Zr—N22—C132	59.71 (18)	C142—C152—C162—C172	−178.5 (3)
O12—Zr—N22—C132	166.0 (2)	C151—C161—C171—C181	0.2 (5)
N11—Zr—N22—C132	111.77 (19)	C151—C161—C171—Cl31	−178.4 (2)
N12—Zr—N22—C132	−19.36 (18)	C152—C162—C172—C182	−2.7 (5)
N21—Zr—N22—C132	−144.68 (18)	C152—C162—C172—Cl32	177.1 (2)
O22—Zr—N22—C142	−140.67 (18)	C161—C171—C181—C191	−1.7 (5)
O21—Zr—N22—C142	132.15 (19)	Cl31—C171—C181—C191	176.8 (2)
O11—Zr—N22—C142	−70.68 (18)	C162—C172—C182—C192	1.7 (4)
O12—Zr—N22—C142	35.64 (18)	Cl32—C172—C182—C192	−178.1 (2)
N11—Zr—N22—C142	−18.6 (2)	C172—C182—C192—C202	1.5 (4)
N12—Zr—N22—C142	−149.8 (2)	C172—C182—C192—Cl42	−179.5 (2)
N21—Zr—N22—C142	84.9 (2)	C171—C181—C191—C201	2.5 (4)
Zr—O11—C11—C21	139.5 (3)	C171—C181—C191—Cl41	−177.6 (2)
Zr—O11—C11—C61	−40.5 (4)	Zr—O21—C201—C191	149.1 (2)
Zr—O22—C12—C22	172.4 (2)	Zr—O21—C201—C151	−31.4 (5)
Zr—O22—C12—C62	−9.4 (5)	C181—C191—C201—O21	177.9 (3)
O11—C11—C21—C31	178.3 (3)	Cl41—C191—C201—O21	−2.1 (4)
C61—C11—C21—C31	−1.8 (4)	C181—C191—C201—C151	−1.6 (4)
O11—C11—C21—Cl11	−0.1 (4)	Cl41—C191—C201—C151	178.4 (2)
C61—C11—C21—Cl11	179.8 (2)	C161—C151—C201—O21	−179.5 (3)
O22—C12—C22—C32	176.0 (3)	C141—C151—C201—O21	0.2 (4)
C62—C12—C22—C32	−2.3 (4)	C161—C151—C201—C191	0.0 (4)
O22—C12—C22—Cl12	−2.8 (4)	C141—C151—C201—C191	179.6 (3)
C62—C12—C22—Cl12	179.0 (2)	Zr—O12—C202—C192	137.0 (2)
C11—C21—C31—C41	1.9 (5)	Zr—O12—C202—C152	−44.3 (4)
Cl11—C21—C31—C41	−179.7 (2)	C182—C192—C202—O12	175.4 (3)
C12—C22—C32—C42	0.7 (4)	Cl42—C192—C202—O12	−3.6 (4)
Cl12—C22—C32—C42	179.4 (2)	C182—C192—C202—C152	−3.4 (4)
C21—C31—C41—C51	−0.2 (5)	Cl42—C192—C202—C152	177.6 (2)
C21—C31—C41—Cl21	179.4 (2)	C162—C152—C202—O12	−176.5 (3)
C22—C32—C42—C52	1.1 (4)	C142—C152—C202—O12	2.7 (4)
C22—C32—C42—Cl22	−177.0 (2)	C162—C152—C202—C192	2.3 (4)
C31—C41—C51—C61	−1.5 (5)	C142—C152—C202—C192	−178.5 (3)

### Hydrogen-bond geometry (Å, °)

**Table:**

*D*—H···*A*	*D*—H	H···*A*	*D*···*A*	*D*—H···*A*
N11—H11···O22	0.89 (2)	2.28 (3)	2.662 (3)	105.7 (18)
N21—H21···O12	0.89 (2)	2.32 (4)	2.722 (4)	108 (2)
N21—H21···Cl42	0.89 (2)	2.76 (2)	3.631 (3)	166.8 (16)
N22—H22···O21	0.89 (2)	2.34 (3)	2.701 (4)	104.1 (18)
C72—H72B···O21	0.99	2.44	3.024 (4)	117
C132—H13B···O11	1.00	2.53	2.987 (3)	108
C141—H14B···O22	0.99	2.58	3.137 (4)	115
C51—H51A···Cl22^i^	0.95	2.83	3.654 (3)	146
C122—H12C···Cl22^ii^	0.99	2.72	3.402 (3)	126

Symmetry codes: (i) −*x*, *y*+1/2, −*z*; (ii) −*x*+1, *y*+1/2, −*z*+1.
